# Metformin‐induced autophagy and irisin improves INS‐1 cell function and survival in high‐glucose environment via AMPK/SIRT1/PGC‐1α signal pathway

**DOI:** 10.1002/fsn3.1006

**Published:** 2019-04-02

**Authors:** Qingxue Li, Shaohui Jia, Lei Xu, Biao Li, Ning Chen

**Affiliations:** ^1^ Tianjiu Research and Development Center for Exercise Nutrition and Foods, Hubei Key Laboratory of Sport Training and Monitoring, College of Health Science Wuhan Sports University Wuhan China; ^2^ School of Sports and Health Linyi University Linyi China; ^3^ Graduate School Wuhan Sports University Wuhan China

**Keywords:** AMPK/SIRT1/PGC‐1α signal pathway, apoptosis, autophagy, INS‐1, irisin

## Abstract

In order to explore the protective function of metformin on pancreatic β cells to alleviate insulin resistance and underlying mechanisms, INS‐1 cells were cultured into normal control (N), high glucose (H), high glucose and metformin (H + Met), high glucose and chloroquine (H + CQ), and high glucose and Ex527 (H + Ex527) groups, respectively. Upon 24‐hr cultivation, the proliferation and glucose‐stimulated insulin secretion (GSIS) of INS‐1 cells were determined, and the expression of irisin and other proteins associated with AMPK/SIRT1/PGC‐1α signal pathway, autophagy, and apoptosis was evaluated. Compared with the N group, the cells from the H group revealed lower proliferation, GSIS, and expression of irisin and proteins associated with AMPK/SIRT1/PGC‐1α signal pathway and autophagy, but higher expression of proteins associated with apoptosis; in contrast, metformin could significantly rescue lower cell proliferation, GSIS, and expression of proteins associated with AMPK/SIRT1/PGC‐1α signal pathway and autophagy, as well as irisin, and suppress apoptosis in high‐glucose environment. Meanwhile, autophagy inhibitor CQ and SIRT1 inhibitor Ex527 can block above functions of metformin. Therefore, metformin can promote INS‐1 cell proliferation, enhance GSIS, and suppress apoptosis by activating AMPK/SIRT1/PGC‐1α signal pathway, up‐regulating irisin expression, and inducing autophagy in INS‐1 cells in high‐glucose environment.

## 1 INTRODUCTION

Diabetes mellitus is a chronic metabolic syndrome with the characteristics of insulin resistance or hyperglycemia caused by absolute or relative insufficiency of insulin secretion and/or deficient function of islet β cells. According to its pathogenesis, diabetes mellitus can be divided into type I diabetes mellitus (T1DM) and type II diabetes mellitus (T2DM) (Thent, Das, & Henry, 2013). Islet β cells are in charge of the secretion of insulin and play an important role in controlling glucose homeostasis. Absolute or relative lacking of islet β cells will eventually lead to the occurrence of diabetes mellitus. Therefore, elucidating molecular mechanisms for regulating the function of islet β cells will have a unique significance for the prevention and treatment of diabetes. Metformin is a widely used drug of T2DM, and its antidiabetic characteristics should be inhibiting hepatic gluconeogenesis and improving insulin sensitivity in peripheral tissues (Adak, Samadi, Unal, & Sabuncuoglu, 2018). Based on previous in vivo studies, metformin also can increase glucose oxidation in MIN6 pancreatic β cells, promote insulin release in mouse isolated pancreatic islets (Hashemitabar, Bahramzadeh, Saremy, & Nejaddehbashi, 2015), and alleviate functional, biochemical, and ultrastructural abnormalities in human islet cells exposed to glucotoxic condition. But so far, the effect and underlying mechanisms of metformin on β cells remain unclear.

Autophagy is a highly conserved process to eliminate aged and miss‐folded proteins or damaged organelles for maintaining cellular homeostasis and promoting cell survival (Levine & Kroemer, 2008,2009; Zhang & Chen, 2018). The dysfunction of autophagy may induce a series of diseases, such as diabetes mellitus, cancer, aging‐related diseases, and neurodegenerative diseases (Chen & Karantza, 2011; Chen & Karantza‐Wadsworth, 2009; Fan et al., 2016; Kou & Chen, 2017; Yang et al., 2017). Deficient function of islet β cells is a major pathological features of diabetes mellitus, but autophagy plays an important role in regulating the structure and functions of β cells through clearing senescent cells and damaged proteins or organelles, and protecting islet β cells against apoptosis (Las & Shirihai, 2010). However, the protective role and the induced autophagy of metformin, as well as corresponding underlying mechanisms in islet β cells, still need to be further investigated.

Maintaining energy homeostasis is one of the major strategies for preventing and treating diabetes mellitus and other metabolic syndromes. AMP‐activated protein kinase (AMPK), as a highly conserved serine/threonine kinase, plays a major role in the regulation of energy metabolism though modulating various regulatory signal pathways (Li, Zhong, Wang, & Zhu, 2017). The reduced expression and activity of AMPK are common in skeletal muscle and fat tissues of animal models with obesity, T2DM, and metabolic syndromes; therefore, either physiological or pharmacological activation of AMPK can mitigate insulin resistance and execute the prevention and treatment of diabetes mellitus (Ruderman, Carling, Prentki, & Cacicedo, 2013). Currently, some metabolic stimuli, such as regular exercise and energy restriction, can induce the increase of AMP/ATP ratio, thus activating AMPK and executing important roles in the treatment of diabetes mellitus through regulating autophagy or myokines (Chen, Li, Liu, & Jia, 2016; Dunstan, 2011; Li, Fan, & Chen, 2018; Yang et al., 2016). Metformin can accomplish the therapeutic efficacy of diabetes mellitus by activating AMPK (Ruderman et al., 2013) and inducing autophagy in distinct tissues (Yao, Zhang, & Chen, 2016). For islet β cells, the protective effect of AMPK is also correlated with its functional induction of autophagy, and the deficient or inhibited AMPK can impair autophagy and accelerate the apoptosis of islet β cells (Wu, Wu, Yang, Wei, & Zou, 2013). Because many contradictions regarding the protective effect of metformin‐induced AMPK on islet β cells are reported (Jiang et al., 2014), its molecular mechanisms need to be further explored and clarified.

Sirtuin 1 (SIRT1), a NAD^+^‐dependent class III histone deacetylase, can result in the deacetylation of peroxisome proliferator‐activated receptor gamma coactivators‐1α (PGC‐1α), p53, forkhead box O (FOXO), nuclear factor kappa‐light‐chain‐enhancer of activated B cells (NF‐κB), and other transcription factors, thereby regulating a series of cellular processes including gene silencing, energy metabolism, antistress, cell survival, and DNA damage repairing (Dong, 2012). SIRT1 involves the regulation of insulin gene transcription and glucose‐stimulated insulin secretion (GSIS) in pancreatic β cells and also protects β cells against inflammation‐induced apoptosis (Lee et al., 2009). So the declined expression and activity of SIRT1 may affect insulin secretion and induce β‐cell apoptosis, thus correspondingly resulting in T2DM. As a multifunctional regulatory factor, PGC‐1α regulates a variety of cellular processes including mitochondrial biogenesis, gluconeogenesis, glucose transport, fatty acid oxidation, peroxisome remodeling, oxidative phosphorylation, and muscle fiber‐type transformation (Corona & Duchen, 2015). Decreased expression of PGC‐1α in rodent peripheral organs including liver, muscle, and adipose tissue is associated with insulin resistance and glucose intolerance (Sczelecki et al., 2014). Islets from human T2DM or β cells cultured in high‐glucose medium usually have reduced expression of PGC‐1α and damaged GSIS (Ling et al., 2008), suggesting the important regulatory role of PGC‐1α on β‐cell function. Therefore, the restoration of PGC‐1α activity may provide an inspiring strategy for the treatment of T2DM.

Currently, a series of circulating factors for inducing compensatory increase of islet β‐cell number are observed to maintain normal function of islet β cells in T2DM (El Ouaamari et al., 2013). Irisin, a newly discovered myokine, is released after the hydrolysis of fibronectin type III domain‐containing protein 5 (FNDC5) regulated by PGC‐1α and plays important roles in regulating energy metabolism (Bostrom et al., 2012; Chen et al., 2016). Irisin can be detected in skeletal muscle, peripheral nerve (epineurium) axons, myelin, testis, pancreas, liver, spleen, and stomach, suggesting that irisin may play a critical role in regulating metabolism in these tissues (Aydin et al., 2014). But so far, the regulation and underlying mechanisms of metformin on irisin are less reported.

Based on the functions of autophagy and the important roles of AMPK/SIRT1/PGC‐1α signal pathway in the regulation of islet β cells (Chen et al., 2013), we make a hypothesis that the protective effect and corresponding mechanisms of metformin on pancreatic β cells may be correlated with induced autophagy and up‐regulated irisin through activating AMPK/SIRT1/PGC‐1α signal pathway. In the present study, INS‐1 cells were cultured in the environments with normal glucose, high glucose, high glucose combined with metformin, high glucose combined with autophagy inhibitor chloroquine, and high glucose combined with SIRT1 inhibitor Ex527, respectively. The expression of proteins associated with AMPK/SIRT1/PGC‐1α signal pathway, autophagy, and apoptosis was evaluated to validate our hypothesis.

## 2 MATERIALS AND METHODS

### 2.1 Chemicals and reagents

RPMI 1640 medium, fetal bovine serum, penicillin, and streptomycin were purchased from Gibco (Gibco, New York, NY, USA). HEPES, l‐Glutamine, β‐mercaptoethanol, and Krebs–Ringer bicarbonate buffer (KRB, pH 7.2) were purchased from Sigma‐Aldrich (St. Louis, MO, USA). Sodium pyruvate was purchased from Sinopharm Chemical Reagent Co., Ltd. (Shanghai, China). Insulin ELISA kit was purchased from Shanghai LAN Biological Technology Co., Ltd. (Shanghai, China). Cell Counting Kit‐8 (CCK‐8) was purchased from Dojindo (Dojindo, Kumamoto, Japan). RIPA buffer and phenylmethylsulfonyl fluoride (PMSF) were purchased from Beyotime Institute of Biotechnology (Jiangsu, China). BCA kit was purchased from Walterson Biotechnology Inc. (Beijing, China). Primary antibodies including phosphor‐AMPK, AMPK, SIRT1, Atg7, p62, Beclin1, LC‐3, Bcl‐2, and Bax (1:1,000), as well as GAPDH (1:10,000), were purchased from Cell Signaling Technology (Danvers, MA, USA). PGC‐1α (1:1,000) and irisin (1:1,000), as well as horseradish peroxidase‐conjugated secondary antibody (1:10,000), were purchased from Abcam (Cambridge, MA, USA). Chemiluminescence (ECL) reagent was purchased from Thermo Scientific (NH, USA).

### 2.2 Cell culture

INS‐1 cells (No. CRB‐130515) were purchased from Saiqi Biological Engineering Co., Ltd. (Shanghai, China) and cultured in RPMI‐1640 medium containing 11.1 mmol/L glucose supplemented with 10% fetal bovine serum (FBS), 100 U/ml penicillin, 100 μg/ml streptomycin, 10 mmol/L HEPES, 2 mmol/L l‐Glutamine, 1 mmol/L sodium pyruvate, and 50 μmol/L β‐mercaptoethanol in an atmosphere of 5% CO_2_ at 37°C, as described previously (Liu, Liu, et al., 2015). According to the experimental design, the cells were cultured in six‐well plates for 24 hr, and then, primary culture medium was replaced with normal culture medium (RPMI‐1640 medium with 11.1 mmol/L glucose, N), high‐glucose culture medium (RPMI‐1640 medium with 33.3 mmol/L glucose, H), metformin (RPMI‐1640 medium with 33.3 mmol/L glucose and 5 μmol/L metformin, H + Met), chloroquine (RPMI‐1640 medium with 33.3 mmol/L glucose and 5 μmol/L chloroquine, H + CQ), and SIRT1 inhibitor Ex527 (RPMI‐1640 medium with 33.3 mmol/L glucose and 0.1 μmol/L Ex527, H + Ex527), respectively, for another 24‐hr cultivation.

### 2.3 Cell proliferation assay

The cells were seeded in 96‐well plates at the density of 10^5^ cells/well for 24 hr and then treated with different prepared medium for another 24‐hr cell cultivation. CCK‐8 kit was used to determine cell proliferation according to the manufacturer's instructions. The cell survival rate = (*A*_experiment_ − *A*_blank_)/(*A*_control_ − *A*_blank_) × 100％, where *A*_experiment_, *A*_blank_, and *A*_control_ were the absorbance at 450 nm from the experimental group, blank group, and control group, respectively.

### 2.4 GSIS test

The cells were seeded in 96‐well plates at the density of 10^5^ cells/well for 24 hr and then treated with different prepared medium for another 24‐hr cell cultivation. After 24‐hr cultivation, the cells were incubated with KRB buffer (pH 7.2) for 1 hr, and then removed the buffer and incubated in KRB buffer with 2.8 mmol/L glucose (low glucose) at 37°C for 1 hr. The supernatant was collected. Then, the cells were incubated with KRB containing 16.7 mmol/L glucose (high glucose) for 1 hr and the supernatant was collected again. The insulin in the supernatant was measured by insulin ELISA kit. The insulin‐releasing stimulation index (ISI) = GSIS/BIS, GSIS, and basal insulin secretion (BIS) are the concentrations of insulin in KRB buffer containing 16.7 and 2.8 mmol/L glucose, respectively (Liu, Liu, et al., 2015).

### 2.5 Western blot analysis

After GSIS test, 3 ml of precooling PBS was added to six‐well culture plate and washed two times. Then, 200 μl of RIPA buffer containing PMSF was added to each well and lysed on ice for 5 min. After blowing and collecting, the cells were subjected to homogenization, ultrasonic treatment, and centrifugation at 6,700 *g* for 10 min at 40°C. Then, the supernatant was collected and total protein concentration was measured by BCA kit. All proteins were sequentially subjected to the denaturation in the presence of sample buffer at 95°C water bath for 5 min, and 20 μg of total protein was loaded and separated by sodium dodecyl sulfate–polyacrylamide gel electrophoresis (SDS‐PAGE), transferred to nitrocellulose membrane, and incubated with appropriate primary antibodies including phosphor‐AMPK, AMPK, SIRT1, PGC‐1α, irisin, Atg7, p62, Beclin1, LC‐3, Bcl‐2, Bax, and GAPDH (1:1,000), and horseradish peroxidase‐conjugated secondary antibody (1:10,000), and then visualized by ECL reagent and imaged by ultrasensitive fluorescence/chemiluminescence imaging system ChemiScope6300 (CLiNX Science Instruments, Shanghai, China).

### 2.6 Statistical analysis

The biochemical parameters were expressed as mean ± standard deviation (*M* ± *SD*). SPSS 17.0 curative statistical processing software (IBM SPSS Inc., Chicago, IL, USA) was used for statistical analysis. One‐way ANOVA was used for the single‐factor analysis, and the statistically significant difference was considered at *p* < 0.05.

## 3 RESULTS

### 3.1 High glucose reduced INS‐1 cell proliferation

**Figure 1:**
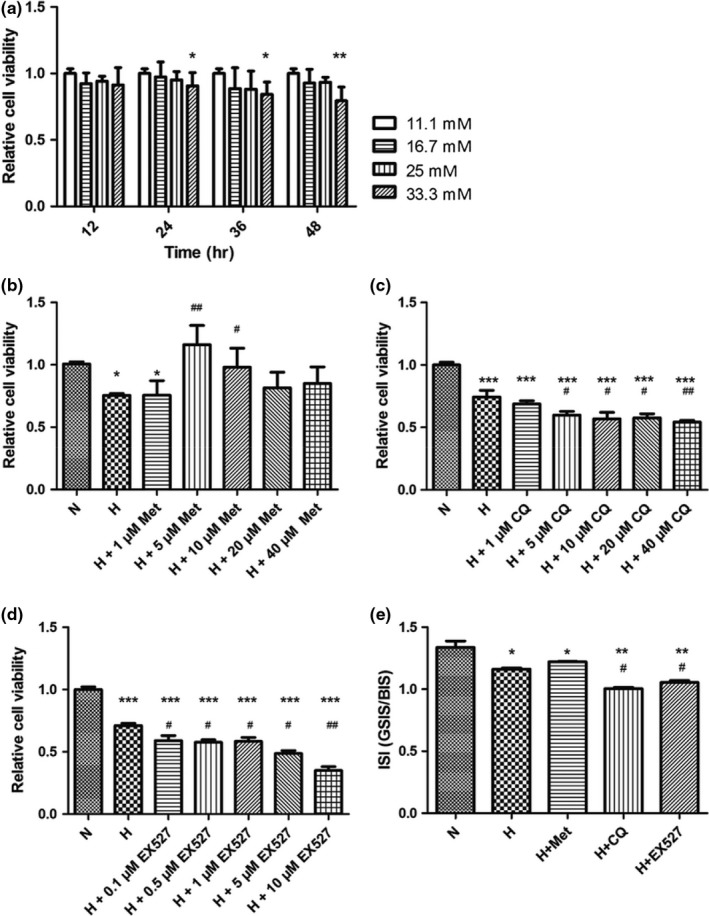
Effects of interventions using high glucose (a) and different drug concentrations (b, c, and d) on cell proliferation of INS‐1 cells were evaluated by Cell Counting Kit‐8 (CCK‐8) kit. Insulin‐releasing stimulation index (ISI) was enhanced by metformin and reduced by high glucose, chloroquine, and Ex527 (e). H + CQ: high glucose culture with 5 μmol/L chloroquine; H + Ex527: high glucose culture with 0.1 μmol/L Ex527; H + Met: high glucose culture with 5 μmol/L metformin; H: high glucose culture; N: normal glucose culture medium. The ISI = GSIS/BIS, GSIS, and BIS represented the concentration of insulin in Krebs–Ringer bicarbonate (KRB) buffer containing 16.7 and 2.8 mmol/L glucose, respectively. *p < 0.05 compared with the N group and ^#^ *p* < 0.05 compared with the H group

INS‐1 cells were cultured in RPMI‐1640 medium containing 11.1, 16.7, 25, and 33.3 mmol/L glucose for 12, 24, 36, and 48 hr, respectively. As shown in Figure 1a, INS‐1 cell proliferation revealed a significant decline when cultured in the medium containing 33.3 mmol/L glucose for 24 hr, suggesting the optimal glucose concentration at 33.3 mmol/L for the model establishment.

### 3.2 Effects of drug types and concentrations on the proliferation of INS‐1 cells

In order to screen the optimal concentration, INS‐1 cells were divided into normal control group (N group), high glucose group (H group), high glucose and metformin group (H + Met group), high glucose and chloroquine group (H + CQ group), and high glucose and Ex527 group (H + Ex527 group). The cells in the N and H groups were cultured in RPMI‐1640 medium containing 11.1 and 33.3 mmol/L glucose, respectively. The cells in the H + Met and H + CQ groups were cultured in high‐glucose medium containing 1, 5, 10, 20, and 40 μmol/L metformin or chloroquine. The cells in the H + Ex527 group were cultured in high‐glucose medium containing 0.1, 0.5, 1, 5, and 10 μmol/L Ex527. Then, INS‐1 cell proliferation rate was estimated by CCK‐8 kit after 24‐hr intervention. As shown in Figure 1b–d, the proliferation of INS‐1 cells revealed a significant decrease in the H group when compared with the N group (*p* < 0.05). Cell proliferation revealed an initial increase and a sequential decrease with the increase of metformin concentration in the H + Met group, and a significant increase at the metformin concentration of 5 and 10 μmol/L when compared with the H group. In contrast, cell proliferation was reduced progressively along with the increase of CQ or Ex527 concentration and revealed the significant reduction at CQ and EX527 concentration of 5 and 0.1 μmol/L, respectively. Therefore, high glucose containing 5 μmol/L metformin, 5 μmol/L CQ, and 0.1 μmol/L Ex527 was selected for the following experiments.

### 3.3 Reduced ISI from high glucose, chloroquine, and Ex527 stimulation was rescued by metformin

INS‐1 cells were subjected to 24‐hr cultivation in H, H + Met, H + CQ, and H + Ex527 medium, respectively. GSIS was estimated by insulin ELISA kit. As shown in Figure 1e, high glucose resulted in the significant reduction of SIS when compared with the N group (*p* < 0.05), and more reduction during combinatorial treatment with CQ and Ex527 (*p* < 0.01). Although no significant difference in the H + Met group and the H group, the increasing trend of SIS could be observed in the H + Met group. Therefore, high glucose, inhibited autophagy, and reduced SIRT1 activity can reduce SIS of INS‐1 cells; in contrast, metformin can rescue the reduced ISI of INS‐1 cells to some extents. Considering chloroquine (Liu, Liu, & Yu, 2016) and Ex527 (Wu et al., 2012) as the specific inhibitor for autophagy and SIRT1, and metformin can prevent from apoptosis through activating AMPK (Jung, Lee, Lee, & Kim, 2012), we hypothesized that the alteration of INS‐1 cell survival and function in high‐glucose environment was correlated with AMPK/SIRT1/PGC‐1α signal pathway and autophagy. Thus, the change in the expression of proteins associated with AMPK/SIRT1/PGC‐1α signal pathway, autophagy, and apoptosis was also evaluated.

### 3.4 Reduced autophagy and enhanced apoptosis induced by high glucose, chloroquine, and Ex527 were rescued by metformin

**Figure 2:**
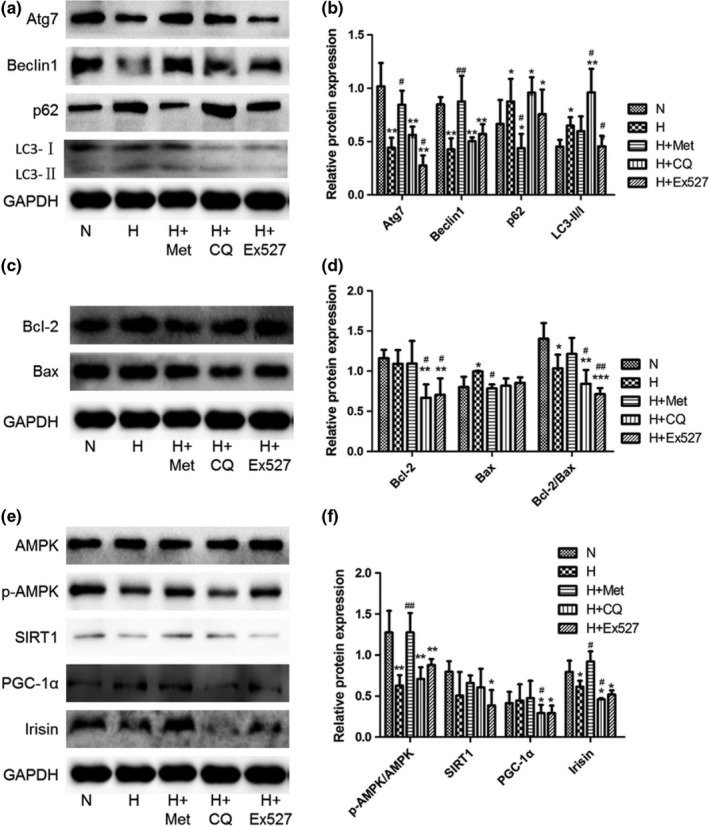
The expression of the proteins associated with autophagy (a and b), apoptosis (c and d), and AMPK/SIRT1/PGC‐1α signal pathway (e and f). The expression of proteins was evaluated by Western blot (a, c, and e), and relative expression of each protein was statistically analyzed by optical density of bands (b, d, and f). The data were expressed as mean ± standard deviation (*M* ± *SD*) from independent experiments performed in triplicate. *p < 0.05, **p < 0.01, and ***p < 0.001 represented the significant difference, very significant difference, and every highly significant difference when compared with the N group; ^#^ *p* < 0.05, ^##^ *p* < 0.01, and ^###^ *p* < 0.001 represented the significant difference, very significant difference, and every highly significant difference when compared with the H group

In order to verify our hypothesis, the expression levels of the proteins associated with autophagy and apoptosis in each group were evaluated. As shown in Figure 2a,b, Atg7 and Beclin1 in INS‐1 cells from the H, H + CQ, and H + Ex527 groups were down‐regulated, while p62 was up‐regulated when compared with the normal group, which was reversed by metformin. In addition, based on the increased LC3‐II/LC3‐I ratio and p62 in INS‐1 cells from the H and H + CQ groups, and down‐regulated p62 in INS‐1 cells in the presence of metformin, metformin can remove denatured or miss‐folded proteins for maintaining cellular homeostasis through regulating dysfunctional autophagy or abnormal autophagy flux.

Similarly, the expression levels of Bcl‐2 and Bax were also determined. Bcl‐2 exhibited an obviously lower expression level in INS‐1 cells from the H + CQ and H + Ex527 groups when compared with the H and H + Met groups, and Bax revealed the lowest expression level in the H + Met group when compared with other groups. Bcl‐2/Bax ratio revealed a significant decrease in INS‐1 cells from the H, H + CQ, and H + Ex527 groups, which was rescued by metformin, as shown in Figure 2c,d. These results suggest that inducing autophagy can suppress high‐glucose‐induced apoptosis of INS‐1 cells.

### 3.5 AMPK/SIRT1/PGC‐1α signal pathway was involved in metformin‐mediated autophagy and apoptosis of INS‐1 cells

In order to explore whether inducing autophagy and suppressing apoptosis of INS‐1 cells was correlated with the activation of AMPK/SIRT1/PGC‐1α signal pathway, the expression levels of proteins including SIRT1, p‐AMPK, AMPK, PGC‐1α, and irisin were evaluated by Western blot. As shown in Figure 2e,f, the reduced p‐AMPK/AMPK ratio and the expression levels of SIRT1 and irisin in INS‐1 cells from the H group were enhanced significantly upon metformin intervention. On the other hand, CQ and Ex527 significantly inhibited the expression of PGC‐1α and irisin in INS‐1 cells cultured under high‐glucose environment. Therefore, the survival and functional improvement of INS‐1 cells exposed to high glucose can be rescued by metformin‐induced autophagy and irisin through regulating AMPK/SIRT1/PGC‐1α signal pathway.

## 4 DISCUSSION

Diabetes has become the major health problem in modern society, which can stimulate cardiovascular diseases and other complications (Ng et al., 2014). The number and function of islet β cells are closely related to the occurrence and development of diabetes, which are reduced under hyperglycemia condition (Calegari et al., 2012). Therefore, the strategies and underlying mechanisms of improving the capacity or function of islet β cells should be explored for the prevention and management of diabetes. As a radiation‐induced rat pancreatic islet tumor cells, INS‐1 cells share similar function for GSIS and stable continuous proliferation capacity of rat pancreatic β cells. Therefore, INS‐1 cell is the optional cell model. In the present study, INS‐1 cells were cultured in RPMI‐1640 medium containing 11.1 mmol/L glucose, and this cultivation environment is consistent with other studies (Kim, Han, & Yoo, 2016). In order to explore the effects of different high‐glucose cultivation systems and cultivation time on INS‐1 cells, we conducted the cultivation of INS‐1 cells in high‐glucose environment and found that INS‐1 cell proliferation was declined with the increase of glucose concentration and the prolongation of culture time. Compared with the normal control group, INS‐1 cell proliferation was declined significantly when cultured in the medium containing 33.3 mmol/L glucose for 24 hr, which is also consistent with previous studies (Tuo, Wang, Li, & Chen, 2011).

Autophagy regulates the function of insulin‐sensitive tissues such as skeletal muscle, liver, visceral fat, and subcutaneous fat, as well as islet β cells (Yang et al., 2017), while deficient autophagy will lead to the impairment of islet β‐cell function and mass, thus eventually leading to the incidence of diabetes (Marrif & Al‐Sunousi, 2016). Several signal pathways involving in the regulation of autophagy, such as AMPK/mTOR signal pathway, have been confirmed to play an important role in protecting islet β cells (Varshney, Gupta, & Roy, 2017). The inhibition of AMPK can result in the impaired autophagy of islet β cells, thus correspondingly leading to excessive apoptosis (Wu et al., 2013), which is also similar with the attenuation of α‐synuclein level due to the induction of autophagy through SIRT1 activation in the chronic MPTP/P‐induced mouse model with Parkinson's disease during treadmill exercise (Koo & Cho, 2017), and the reduced atrophy of skeletal muscle in D‐gal‐induced aging rats during ampelopsin‐induced autophagy through activating AMPK/SIRT1/PGC‐1α signaling cascade (Kou et al., 2017).

AMP‐activated protein kinase is a nutrient and energy sensor for maintaining cellular energy balance, and its inhibited activity is correlated with the occurrence and development of diabetes mellitus. According to this proposal, many medications and treatments including metformin have been developed and widely used for treating T2DM. Several studies have confirmed that metformin for improving blood glucose homeostasis and promoting insulin release is associated with the activation of AMPK (Long & Zierath, 2006). Therefore, the activation of AMPK may represent a therapeutic approach to improve insulin action and prevent a decrease in β‐cell function during the progression of T2DM (Pold et al., 2005). But so far, the protective effect of AMPK on islet β cells is still debated. As a multifunctional transcription factor, SIRT1 involves in mitochondrial biogenesis, glycolipid metabolism, and insulin secretion, thereby resulting in a complex correlation between SIRT1 and AMPK. For example, exogenous cAMP can activate AMPK and SIRT1 and may be related to the anti‐aging effect of calorie restriction (Wang et al., 2015). The reduced insulin secretion stimulated by glucose is observed in SIRT1 knockout mice (Bordone et al., 2006), and the overexpression of SIRT1 gene can result in the declined blood cholesterol and fasting blood glucose, and increased insulin sensitivity in SIRT1 transgenic mice (Bordone et al., 2007). Similarly, the overexpression of SIRT1 alleviates the toxicity of RIN cells and reduces nitric oxide (NO) production and the expression of inducible nitric oxide synthase (iNOS) (Lee et al., 2009). SIRT1 can also directly bind to uncoupling protein 2 (UCP2) promoter and inhibit the expression of UCP2 gene in INS‐1 cells, thus improving insulin secretion of islet β cells. Moreover, the inhibited expression of SIRT1 upon siRNA can increase UCP2 level, block insulin secretion, and limit ATP generation after glucose stimulation. Therefore, SIRT1 regulates insulin secretion by regulating the expression of UCP2 in β cells (Chen et al., 2013). During exploring the interactions and functions of SIRT1 and its target proteins in the development of insulin resistance, the reduced expression of SIRT1 and PGC‐1α is observed in pancreatic β cells of Sprague Dawley (*SD*) rats with insulin resistance induced by high‐glucose and high‐fat diet, while caloric restriction stimulates the expression of SIRT1 and PGC‐1α. Thus, SIRT1 and its downstream transcription factor PGC‐1α may play a protective effect on islet β cells in rat pancreas (Chen et al., 2013). Irisin can significantly improve glucose tolerance in obese mice and inhibit hepatic gluconeogenesis and increases glycogen synthesis in T2DM and hepatocytes (Liu, Shi, et al., 2015), which provides the new hope of irisin as the target for the prevention and treatment of T2DM. As a downstream regulator of PGC‐1α, the protective function of irisin may be dependent on the activation of AMPK/SIRT1/PGC‐1α signal pathway.

Metformin is a widely used drug in the treatment of T2DM, and its antidiabetic characteristics should be correlated with inhibiting hepatic gluconeogenesis and increasing insulin sensitivity of peripheral tissues (Adak et al., 2018). In vivo studies, metformin increases glucose oxidation in MIN6 pancreatic β cells (Jiang et al., 2014), stimulates insulin release in isolated pancreatic islets from mice (Hashemitabar et al., 2015), and prevents functional, biochemical, and ultrastructural abnormalities in human islet cells exposed to glucotoxic condition. These effects can be critical in the treatment of T2DM (Mousavi et al., 2015). In the present study, metformin rescued GSIS and proliferation of INS‐1 cells exposed to high‐glucose environment. In order to explore the correlation between autophagy and apoptosis on these protective effects of metformin, the expression levels of the proteins associated with autophagy and apoptosis were evaluated in INS‐1 cells intervened with high glucose and various drugs. The results showed that metformin could significantly activate autophagy through up‐regulating Atg7 and Beclin1, and accelerating p62 degradation, thereby significantly inhibiting apoptosis through increasing Bcl‐2/Bax ratio and down‐regulating Bax when compared with the normal control group. So, high‐level glucose inhibited autophagy and induced apoptosis, which was restored by metformin. Recent studies have demonstrated that the activation of AMPK by metformin plays an important role in cell metabolism and improves cell viability and function (Adak et al., 2018). Resveratrol (a SIRT1 activator) also has the characteristics of antidiabetic by increasing energy expenditure, which is regulated by the activity of AMPK, SIRT1, and PGC‐1α (Vetterli, Brun, Giovannoni, Bosco, & Maechler, 2011). In our study, cotreatment with metformin significantly enhanced p‐AMPK/AMPK ratio and protein expression of SIRT1 in high‐glucose‐stimulated INS‐1 cells, suggesting that the activation of AMPK/SIRT1/PGC‐1α signal pathway may exert a protective effect against diabetes. In previous studies, appropriate increase in circulating irisin can rescue insulin sensitivity of C57BL/6 mice with diet‐induced insulin resistance (Lopez‐Legarrea et al., 2014). Similarly, irisin can cause the reduced apoptosis of INS‐1 cells induced by high glucose and the increased secretion of insulin (Liu et al., 2017). In the present study, the significantly decreased irisin was also detected during the exposure to high‐glucose environment, while restored by metformin. Therefore, metformin may play an important role in the maintenance and protection of pancreatic tissues and islet β cells through up‐regulating AMPK/SIRT1/PGC‐1α‐mediated irisin.

In order to further validate the relevance of activating AMPK/SIRT1/PGC‐1α signal pathway, inducing autophagy, and reducing pancreatic islet cell apoptosis, the changes in ISI and protein expression associated with autophagy, apoptosis, and AMPK/SIRT1/PGC‐1α signal pathway were also detected in INS‐1 cells intervened with CQ and Ex527. As an autophagy inhibitor, CQ can hamper the fusion of autophagosomes and lysosomes, and result in the elevated LC3‐II/LC3‐I ratio (Chen, Melchior, Wu, & Guo, 2014). In addition, Ex527, as a selective inhibitor of SIRT1, can inhibit deacetylase activity of SIRT1 effectively. For further exploring the protective effects of autophagy and SIRT1 on INS‐1 cells, CQ and Ex527 were selected and the validation studies were conducted. The results showed that high‐level glucose inhibited cell proliferation, ISI, and autophagy and induced apoptosis, while cotreatment with Ex527 or CQ further inhibited cell proliferation, ISI, and autophagy and induced cell apoptosis. However, impaired autophagy and enhanced apoptosis were restored by metformin, which is consistent with previous studies describing the progressive diminution in the number of islet β cells through apoptosis (Marrif & Al‐Sunousi, 2016), and the deficient autophagy could decrease the number and function of islet β cells (Kim et al., 2014). As for the expression of proteins associated with AMPK/SIRT1/PGC‐1α signal pathway and irisin, cotreatment with Ex527 and CQ markedly inhibited the expression of SIRT1, PGC‐1α, and irisin. Therefore, autophagy mediated by AMPK/SIRT1/PGC‐1α signal pathway may play an important role in the maintenance and protection of pancreatic β cells.

## 5 CONCLUSION

**Figure 3:**
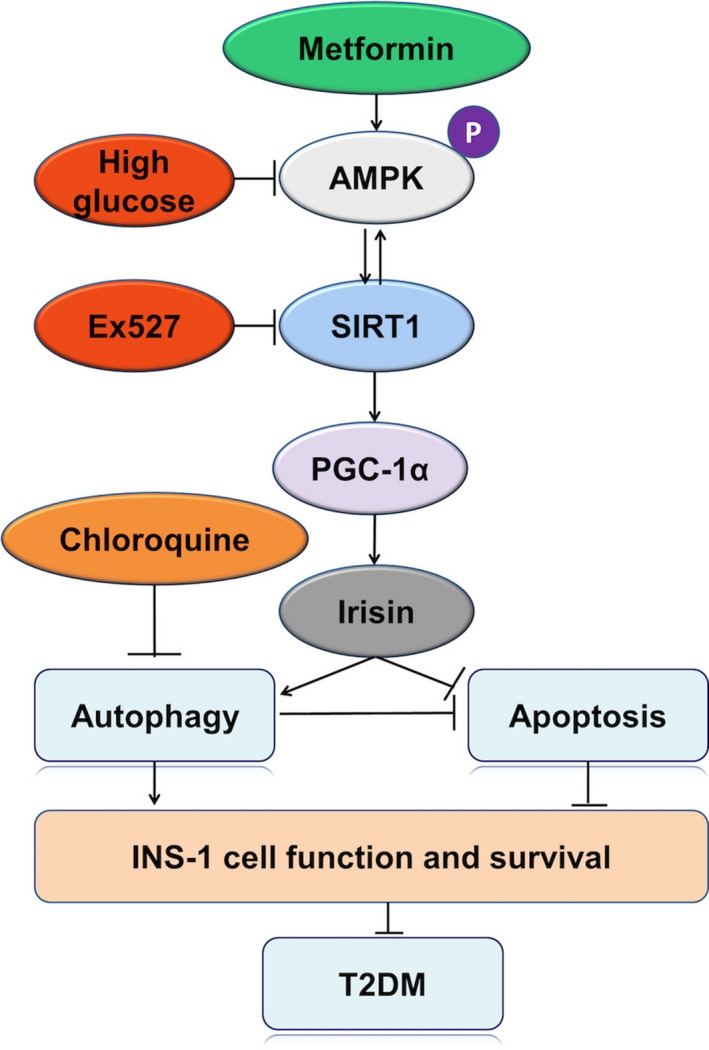
Metformin‐induced autophagy and irisin improves INS‐1 cell function and survival in high‐glucose medium through activating AMPK/SIRT1/PGC‐1α signal pathway. High glucose down‐regulated AMPK/SIRT1/PGC‐1α signal pathway, irisin, and autophagy, induced apoptosis, and reduced INS‐1 cell function and survival, thus inducing diabetes mellitus. Autophagy inhibitor CQ and SIRT1 inhibitor Ex527 could suppress AMPK/SIRT1/PGC‐1α signal pathway, irisin, and autophagy, induce apoptosis, and exacerbate diabetes mellitus

As a widely used antidiabetic drug, metformin may also exert the protective effect on islet β cells from high‐glucose‐induced damage by activating autophagy and up‐regulating irisin via AMPK/SIRT1/PGC‐1α signal pathway (Figure 3), which will provide a novel theoretical evidence that inducing autophagy through activating AMPK/SIRT1/PGC‐1α signal pathway is a potential interventional strategy for the prevention and treatment of diabetes mellitus.

## ETHICAL APPROVAL

This study does not involve any human or animal testing.

## CONFLICT OF INTEREST

These authors have declared no conflict of interest.
